# Microinjection induces changes in the transcriptome of bovine oocytes

**DOI:** 10.1038/s41598-020-67603-4

**Published:** 2020-07-08

**Authors:** Minjie Tan, Helena T. A. van Tol, Michal Mokry, Tom A. E. Stout, Bernard A. J. Roelen

**Affiliations:** 1 Department of Population Health Sciences, Faculty of Veterinary Medicine, Utrecht University, Utrecht, The Netherlands; 2 Epigenomics Facility, University Medical Center Utrecht, Utrecht, The Netherlands; 3 Department of Clinical Sciences, Faculty of Veterinary Medicine, Utrecht University, Utrecht, The Netherlands

**Keywords:** Cell biology, Biological techniques, Sequencing, Developmental biology, Embryology

## Abstract

Gene knockdown techniques are widely used to examine the function of specific genes or proteins. While a variety of techniques are available, a technique commonly used on mammalian oocytes is mRNA knockdown by microinjection of small interfering RNA (siRNA), with non-specific siRNA injection used as a technical control. Here, we investigate whether and how the microinjection procedure itself affects the transcriptome of bovine oocytes. Injection of non-specific siRNA resulted in differential expression of 119 transcripts, of which 76 were down-regulated. Gene ontology analysis revealed that the differentially regulated genes were enriched in the biological processes of ATP synthesis, molecular transport and regulation of protein polyubiquitination. This study establishes a background effect of the microinjection procedure that should be borne in mind by those using microinjection to manipulate gene expression in oocytes.

## Introduction

The generation of gene knockout models based around embryonic stem (ES) cells and chimeric animals has significantly enhanced our knowledge of the function of specific genes. A variety of techniques are available to manipulate or alter the genome including homologous recombination, transcription activator-like effector nucleases (TALENs) and clustered regularly interspaced short palindromic repeats (CRISPR/Cas9)^1–3^.

In oocytes and early stage embryos, gene transcription is arrested in the period between maternal meiotic resumption and embryonic genome activation. In addition, most maternal mRNAs are eliminated soon after the time of embryonic genome activation^4^. In the period of transcriptional arrest, the oocyte and then the blastomeres are dependent on transcripts produced in the oocyte prior to germinal vesicle (GV) breakdown. For this reason, gene knockout techniques are less practical for genes critical to oocyte maturation or embryonic development before embryonic genome activation. Particularly where homozygous gene knockout leads to embryonic lethality, gene function during oocyte development cannot be studied because functional transcripts will be present in heterozygous mothers.

As an alternative, mRNA translation can be downregulated by the introduction of small interfering RNA sequences (siRNA) into oocytes or early embryos^5,6^. In vivo, mRNA expression is regulated by small RNAs with sequences homologous to the target mRNA that use proteins such as DICER to form a multicomponent nuclease, RNA-induced silencing complex (RISC), to eliminate the target mRNA^7^. For species where ES cells are not available, but also for maternally expressed genes, RNA interference (RNAi) can be a useful tool for generating information about gene function. Small RNAs directed against mRNA sequences can be artificially synthesized and introduced into oocytes using electroporation or by direct injection to downregulate mRNA expression^8–10^.

However, genome-wide analysis has revealed that siRNA sequences can also regulate expression of unintended targets^11^. So-called off-target effects can complicate the interpretation of phenotypic effects during gene-silencing experiments^12,13^. Off-target effects can be minimized, but not eliminated, by strategies such as careful selection of the siRNA sequence and the comparison of several different siRNA sequences that target the same mRNA^12, 14^.

When mRNA expression is downregulated by siRNA sequences, nonspecific RNA sequences, preferably of similar size and containing the same nucleotides but in a ‘scrambled’ order to the gene specific siRNA sequence, are used as a negative control. In oocytes and single-cell zygotes the preferred delivery method for siRNA is microinjection. During microinjection the plasma membrane is damaged, cytoskeletal structures may be disrupted, positions of organelles can be changed, and foreign components are introduced into the cell. Although injection of scrambled siRNA is an important control for validating the specificity of siRNA and its targeting for mRNA downregulation, the damaging effects of microinjection and introduction of short RNA sequences per se on the cell have not been analyzed in any depth.

Here we evaluate the effect of injecting small RNA sequences into oocytes, in particular with respect to changes in the transcriptome. Denuded oocytes were injected at the GV stage and cultured first for 16 h in the presence of roscovitine to delay germinal vesicle breakdown and allow more time for the siRNA to exert its influence^15,16^. Single oocyte RNA sequencing (RNA-Seq) was performed to examine changes in RNA expression as a result of the injection procedure.

## Results

### Microinjection does not significantly influence the ability of oocytes to mature in vitro

**Figure 1:**
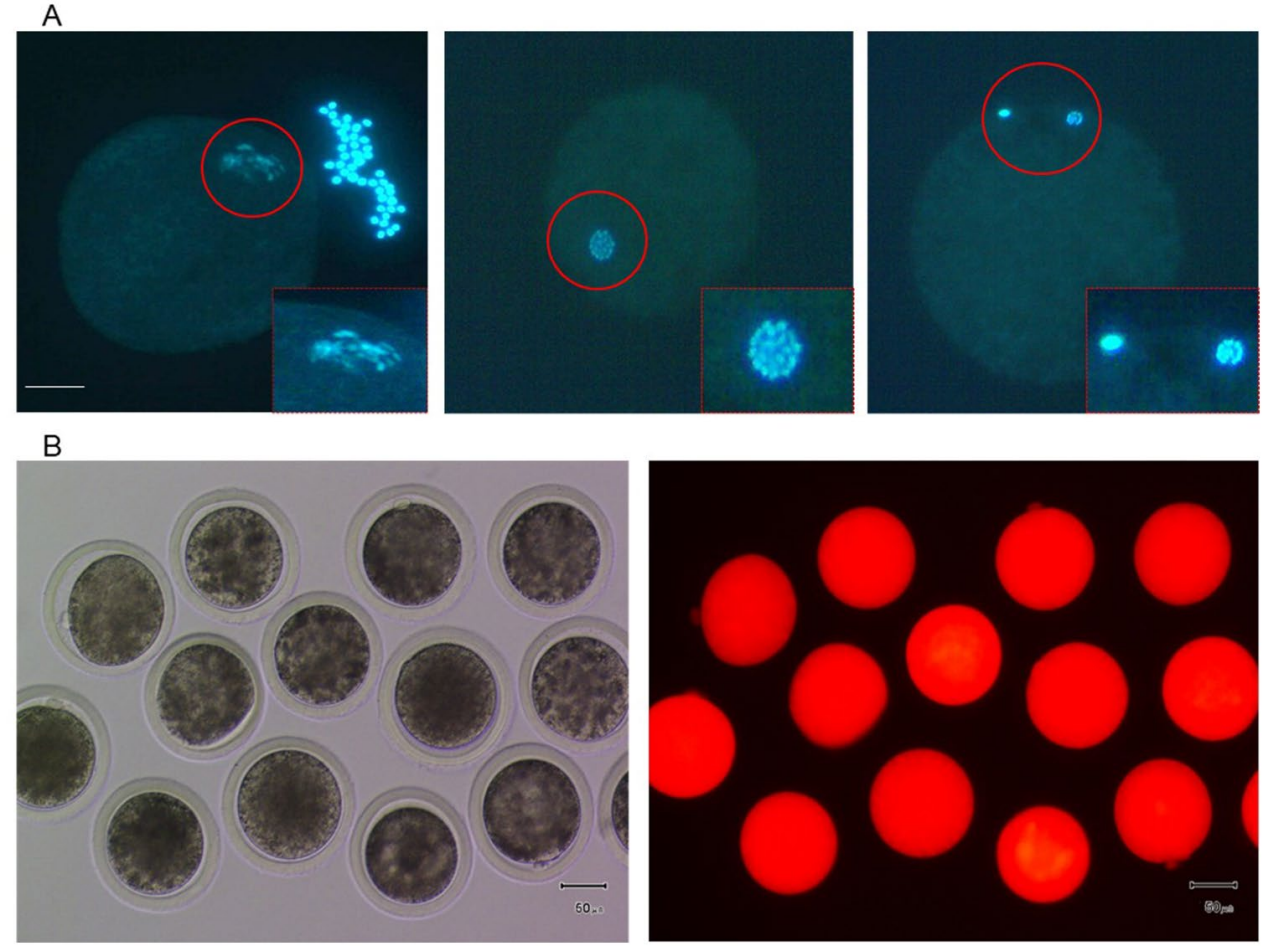
Microinjection of bovine oocytes. Oocytes injected with non-specific siRNA mixed with Dextran-TRITC at the germinal vesicle stage and cultured in roscovitine for 16 h before maturation in vitro. (**A**) Oocytes at germinal vesicle (left), Metaphase I (middle) and Metaphase II (right) stages, based on chromatin distribution (DAPI staining in blue. Insets show magnified view of red encircled areas. Scale bar: 30 μm. (**B**) Bright field (left) and fluorescence (right) microscopic images of matured Metaphase II stage oocytes after siRNA injection. Red fluorescence indicates successful injection. Scale bars: 50 μm.

Denuded GV stage bovine oocytes were microinjected with non-specific siRNA containing Dextran-TRITC, as an indicator of successful injection. Only oocytes that showed red fluorescence were cultured further. Regular in vitro maturation (IVM) of bovine oocytes takes 24 h, which might be too short for the RNAi machinery or any other intervention to properly downregulate mRNA expression; in that case, researchers usually extend the reaction time with chemical inhibitors such as roscovitine to delay in vitro maturation. Therefore, oocytes were first cultured in the presence of the cyclin-dependent kinase (CDK) inhibitor roscovitine for 16 h to delay meiotic resumption, followed by culture in conventional maturation medium for 22 h. After 16 h of culture in roscovitine, most oocytes had reached the metaphase (M)I stage (Fig. 1A). At the end of IVM, injected MII stage oocytes still contained red fluorescence, indicating that the injection procedure had not caused significant leakage (Fig. 1B). The maturation rate (based on the numbers of oocytes with a first polar body) of the siRNA injected group (80.4%) was not different to that of the control group (86.8%, Table 1); this was expected given that an injected siRNA was chosen with little homology to known mRNA sequences. These results demonstrate that the injection procedure per se did not affect the oocyte’s capacity to resume meiosis.

**Table 1:** Non-specific siRNA microinjection does not influence the maturation rates.

	**Non-specific siRNA injection**	**Non-injected**
Oocytes	97	68
Matured	78	59
Percentage matured (NS)	80.4%	86.8%

*Pooled data from 3 replicates. Maturation rate: % of oocytes in metaphase II. *NS* Non significant *p* = 0.29.

### Single oocyte RNA sequencing

**Figure 2:**
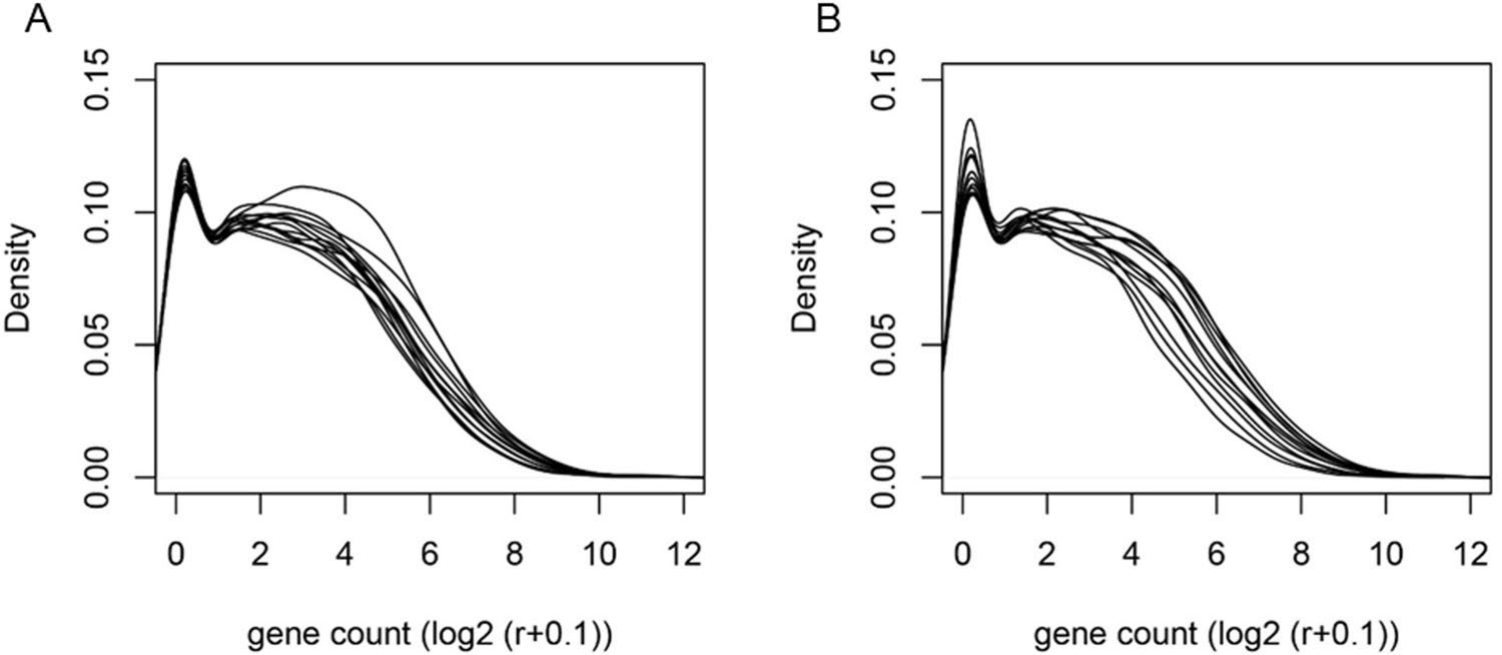
Distribution of expression levels of each single cell transcriptome in (**A**) non-specific siRNA injected and (**B**) non-injected oocytes. Individual lines represent individual oocytes, 12 oocytes per group.

To improve the accuracy of RNA sequencing result and reduce random transcriptome difference based on single oocytes, 12 single oocytes per group (microinjected and non-injected group) were chosen for Cell-seq2^17^ analyze. The expression of each transcript in each sample was normalized with reads per million (RPM). Analyses of RPM values for each replicate showed that the overall ranges and distribution of the RPM values were consistent among the samples (Fig. 2). These results indicate that our RNA-seq data are reliable, reproducible and of high quality, and meet the conditions for differential expression analysis.

### Microinjection alters gene expression

**Figure 3:**
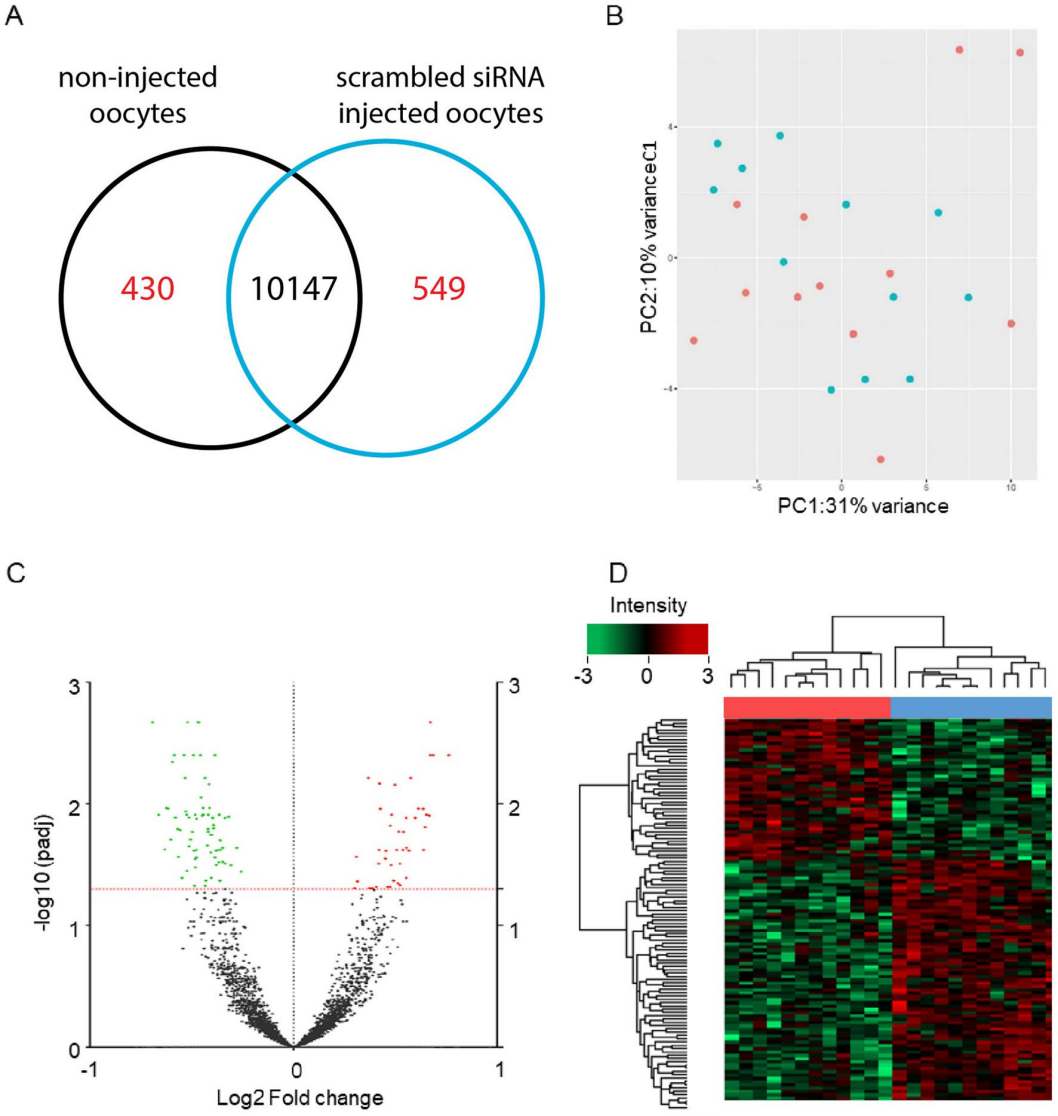
Gene expression differences between non-injected and siRNA injected oocytes. (**A**) Venn diagram showing overlapping and specific transcripts in the two groups. (**B**) Principal component analysis (PCA) of the transcriptomes; PC1 and PC2 represent the top two dimensions of the differentially expressed genes among the groups. Blue: non-injected oocytes; red: injected oocytes. (**C**) Volcano plot showing the estimated fold change (X-axis) versus the –log10 values (Y-axis) for non-injected and siRNA injected oocytes. Red dots represent genes significantly up-regulated in the injected group, green dots represent genes significantly down-regulated in the injected group (adjusted P-value < 0.05). Black dots represent transcripts for which expression did not differ significantly. (**D**) Heatmap showing the results of cluster analysis for differentially expressed genes between the two groups; n = 119 (red: upregulated; green: downregulated). Red bar: injected oocytes. Blue bar: non-injected oocytes.

To analyze the effect of microinjection in oocytes, we compared the gene expression profiles between the injected and non-injected groups. The total numbers of detected genes ranged from 9,153 to 12,094 over the two groups. On average, 10,577 different mRNAs were detected in the non-injected group whereas 10,696 expressed genes were detected in the siRNA injected group. Among these, 10147mRNAs were detected in both groups, while 430 and 549 were detected exclusively in non-injected and siRNA injected oocytes, respectively (Fig. 3A). Principal component analysis (Fig. 3B) revealed only a minor difference between oocytes from non-injected and siRNA injected groups. Nevertheless, 119 transcripts were differently expressed between the two groups based on an adjusted p value < 0.05 with a fold change > 1.2 (Fig. 3C, supplementary Table 1). Of the differently expressed mRNAs, 76 transcripts were down-regulated and 43 transcripts were up-regulated in the injected oocytes (Fig. 3D).

In order to verify that the exclusively expressed genes in the injected oocytes were indeed due to the injection procedure, oocytes were also injected with a combination of 3 specific siRNAs directed against an oocyte-expressed gene (*PIWIL3*) and individually sequenced. Since in this group of oocytes an expressed gene was targeted, the differences in expressed mRNA with non-injected oocytes was larger. Indeed, *PIWIL3* expression in these oocytes was reduced, but the maturation rate was similar to that of control oocytes (Supplementary Fig. 1). Importantly however, of the 549 exclusive mRNAs in non-specific siRNA injected oocytes, 540 (98%) were overlapping with the specific siRNA injected oocytes (Supplementary Fig. 1), confirming that expression of these genes is caused by the injection procedure.

### Microinjection alters the expression of mitochondrial and transmembrane related genes

**Figure 4:**
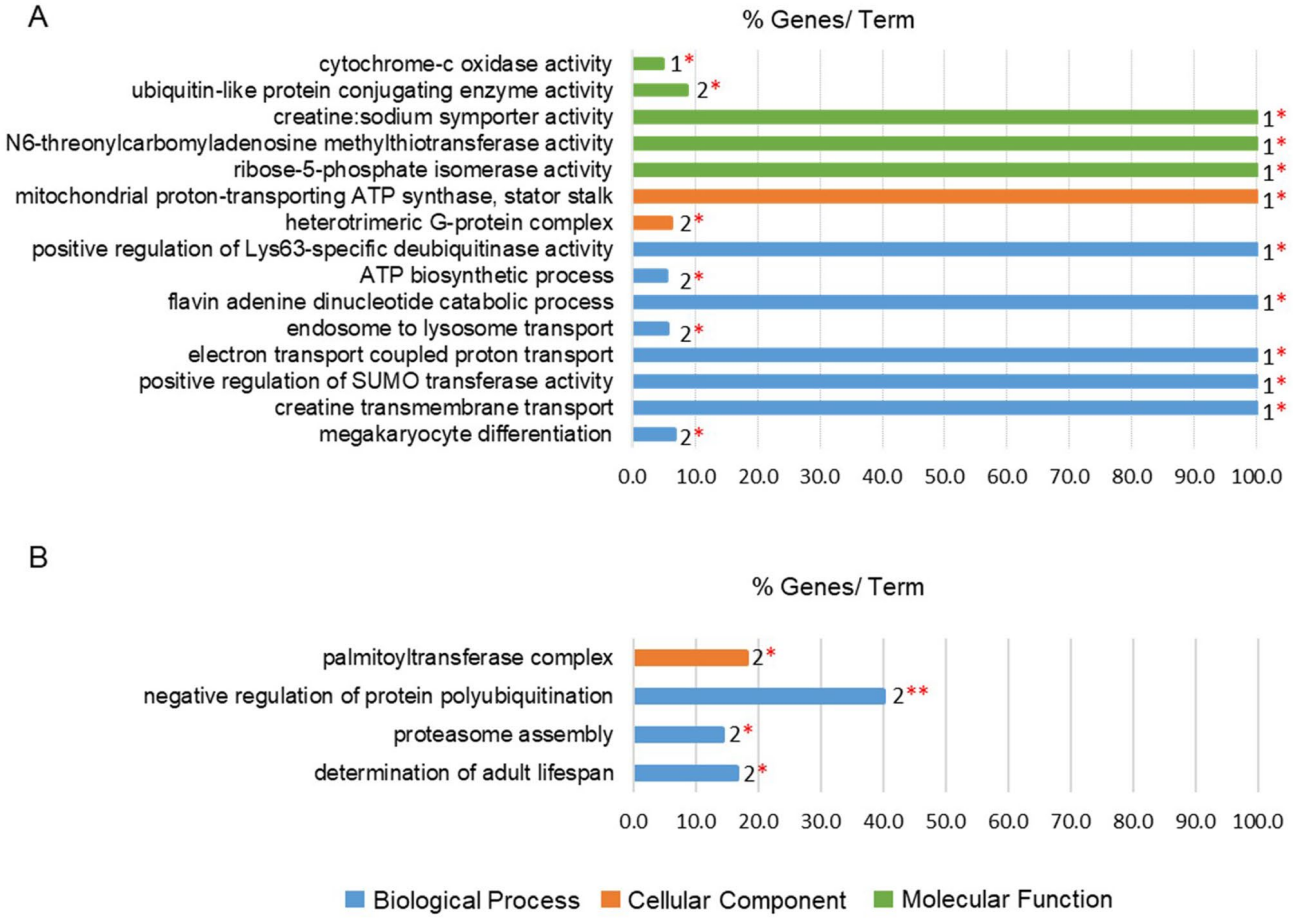
Functionally grouped gene ontology (GO) terms for genes significantly up-regulated/down-regulated in non-specific siRNA injected oocytes compared to non-injected oocytes. (**A**) Up-regulated GO terms in siRNA injected oocytes. (**B**) Down-regulated GO terms in siRNA injected oocytes. Number of genes (number behind bar chart) and the percentage of mapped genes from the total number of genes of the term is shown. GO Term is labeled on the left hand side. The level of significance of the terms is shown as ** (p value < 0.001), * (0.001 < p value < 0.05). Same bar color indicates the Go term as being in the same ontology.

To gain better insight into alteration of gene expression after siRNA injection, Gene Ontology (GO) term enrichment analysis was performed using ClueGO software (Fig. 4, Table 2). We identified ‘biological process, ‘cellular component’ and ‘molecular function’ as being enriched among these differently expressed mRNAs. For up-regulated transcripts, 8 GO terms within ‘biological process’ were significantly enriched and these incorporated 8 genes: *VCP, ATP5H, SNAPIN, COX1, UBE2I, SLC6A8, ABI1 and HMGB2.* Among them, *VCP* was included in 4 different ‘biological process’ lists, namely “positive regulation of Lys63-specific deubiquitinase activity”, “ATP biosynthetic process”, “flavin adenine dinucleotide catabolic process” and “endosome to lysosome transport”. Two cellular component GO terms were significantly enriched, “mitochondrial proton-transporting ATP synthase, stator stalk” which includes *ATP5H*, and “heterotrimeric G-protein complex” which includes *GNA11* and *GNB2*. Six genes (*COX1, UBE2C, UBE2I, SLC6A8, CDKAL1* and *RPIA*) contributed to 5 significantly enriched GO terms for ‘molecular function’ relating to activity of multiple enzymes. For the down-regulated transcripts, 3 GO terms were enriched within ‘biological process’, where “negative regulation of protein polyubiquitination” was the most significant GO term, and related to two genes (*PLAA* and *TRIP12*), while “palmitoyltransferase complex” was the only GO term enriched within ‘cellular component’.

**Table 2:** Lists of functionally grouped gene ontology (GO) terms significantly up-regulated/down-regulated in siRNA injected oocytes compared to non-injected oocytes.

GO_ID	GO_Term	Genes
GO:0004129	Cytochrome-c oxidase activity	[COX1]
GO:0061650	Ubiquitin-like protein conjugating enzyme activity	[UBE2C, UBE2I]
GO:0005309	Creatine:sodium symporter activity	[SLC6A8]
GO:0035598	N6-threonylcarbomyladenosine methylthiotransferase activity	[CDKAL1]
GO:0004751	Ribose-5-phosphate isomerase activity	[RPIA]
GO:0000274	Mitochondrial proton-transporting ATP synthase, stator stalk	[ATP5H]
GO:0005834	Heterotrimeric G-protein complex	[GNA11, GNB2]
GO:1903007	Positive regulation of Lys63-specific deubiquitinase activity	[VCP]
GO:0006754	ATP biosynthetic process	[ATP5H, VCP]
GO:0072389	Flavin adenine dinucleotide catabolic process	[VCP]
GO:0008333	Endosome to lysosome transport	[SNAPIN, VCP]
GO:0015990	Electron transport coupled proton transport	[COX1]
GO:1903755	Positive regulation of SUMO transferase activity	[UBE2I]
GO:1902598	Creatine transmembrane transport	[SLC6A8]
GO:0030219	Megakaryocyte differentiation	[ABI1, HMGB2]

GO_ID	GO_Term	Genes
GO:0002178	Palmitoyltransferase complex	[GOLGA7, SPTLC1]
GO:1902915	Negative regulation of protein polyubiquitination	[PLAA, TRIP12]
GO:0043248	Proteasome assembly	[POMP, PSMG1]
GO:0008340	Determination of adult lifespan	[IDE, MSH2]

*Upper table* Up-regulated GO terms and gene names in siRNA injected oocytes, *Lower table* Down-regulated GO terms and gene names in siRNA injected oocytes.

## Discussion

RNAi has been widely used as a method for suppressing gene expression^18^. For gene expression knockdown before the embryonic genome has been switched on in oocytes and preimplantation embryos , microinjection in oocytes is the preferred method of delivering siRNA, because the timing and amount of introduced RNA can be controlled^19,20^. Alternatively, cell transfection with for instance lipofectamine can be used to introduce siRNA into cells. For efficient delivery using lipofectamine, removal of the zona pellucida is required however, which reduces the developmental competence of the zygote^6^. Also, the timing and amount of siRNA delivery are less precise using transfection using lipofectamine. Electroporation has also been used to successfully introduce small RNAs and protein into preimplantation embryos, and for efficient transfection the pulse number, pulse duration, and voltage strength first need to be established^21^. A disadvantage of microinjection, compared with for instance transfection, is the physical stress that the cells are subjected to as the cell membrane is ruptured and liquid with RNA is deposited into the cytoplasm. Also, the microinjection procedure requires training in use of the equipment. Particularly for gene expression knockdown via siRNA, oocyte injection is however the most used method.

Independent of the delivery method, the RNAi machinery needed for the degradation of mRNA, and natural turnover for downregulation of protein levels, requires a period of time longer than the 22 h during which cattle oocytes normally reach the MII stage when matured in vitro. Oocytes were therefore cultured in the presence of the CDK inhibitor roscovitine for 16 h before being cultured in maturation medium for 22 h^15,22^. To distinguish non-specific from specific effects, scrambled siRNAs are usually injected and compared with oocytes/zygotes injected with specific siRNAs. A possible downside of this approach is that the effects of the injection procedure itself might be masked, but influence the final results.

Oocyte integrity after microinjection has been studied, but primarily to assess the impact of intracytoplasmic sperm injection (ICSI). For ICSI, injected oocytes are usually already at the MII stage; otherwise, the injection procedure is very similar to that required for siRNA injection. After ICSI, ultrastructural changes within oocytes have been reported, and include membrane-bound vacuoles and plasma membrane inclusions^23^. Although with human oocytes a lower blastocyst rate has been reported after ICSI^24^, a meta-analysis study did not indicate differences in birth defects between ICSI and regular IVF born children^25^. In bovine oocytes it has been reported that fewer blastocysts developed from ICSI oocytes than from oocytes that underwent conventional IVF^26^ but it cannot be distinguished whether these differences were the result of the injection procedure or the differences in sperm selection.

Here we analyzed the potential damage of the siRNA microinjection procedure per se by comparing non-injected oocytes with oocytes injected with siRNAs that did not target a specific mRNA. After injection, similar percentages of oocytes matured to the MII stage, indicating that the initial developmental capacity of the oocyte was not altered by the microinjection procedure. We have however not examined the capacity of the injected oocytes to develop to the blastocyst stage after fertilization. When embryos develop after injection, the cryopreservation efficiency may be diminished as has indeed been demonstrated for early embryos derived after DNA microinjection^27^ or after ICSI^28^.

We have examined the transcriptome in individual injected oocytes and compared it to that from non-injected oocytes^17^. Transcriptome analysis of single oocytes has been reported in various species including mouse, cow and man^29–31^. We detected approximately 10,600 genes as being expressed in bovine oocytes, which is similar to what has been reported previously^30,32,33^.

To understand transcriptome and biological pathway changes after siRNA microinjection and to define a background reference effect for conducting future siRNA microinjection experiments, we performed GO analysis based on genes with significantly altered expression levels. Our study identified 11 biological processes, 3 cellular components and 5 molecular functions that were significantly affected by microinjection, based on up and down-regulated genes.

The oocyte membrane and cytoskeleton play important roles in maintaining cell shape, and enabling cytokinesis during cell divisions. During microinjection, the injection needle disrupts the integrity of the plasma membrane and it is therefore not surprising that expression of genes related to transmembrane proteins, cytoskeleton and actin filaments were significantly altered by siRNA microinjection. For instance, *GNA11* and *GNB2* that belong to the heterotrimeric G-protein complex alpha and beta subunits respectively are involved as modulators or transducers in various transmembrane signaling systems. G-alpha subunits are tethered to the plasma membrane and activate the enzyme adenylyl cyclase. ACTR2*,* also known as ARP2 Actin Related Protein 2 Homolog, is part of the ARP2/3 complex that mediates the formation of branched actin networks in the cytoplasm and is important for asymmetric division, spindle migration as well as the formation and completion of oocyte cytokinesis during meiotic maturation^34,35^. The upregulation of *ACTR2 expression* suggests that actin remodeling is required during oocyte maturation after injection. *GOLGA7*, which was down-regulated by siRNA injection codes for the palmitoyltransferase complex and may also be involved in vesicular protein transport from the Golgi apparatus to the cell surface^36^.The downregulation of *GOLGA7* transcripts in our data suggests that injection interrupts signaling between cytoplasm, plasma membrane and organelles.

Mitochondria are essential for multiple biological processes via ATP synthesis in maturing oocytes^37^. Indeed, oocyte quality can be interrupted by mitochondrial dysfunction and inadequate ATP production^38^. *ATP5H* and *VCP* are related to ATP synthesis and were up-regulated in oocytes injected with siRNA, indicating that microinjected oocytes require extra ATP to reach the MII stage.

Ubiquitin is involved in post-translational modification of target proteins. During the maternal to zygotic gene transition, maternal proteins are degraded by the ubiquitin–proteasome system and new proteins are synthesized from the embryonic genome. Reduced proteasome activity leads to accumulation of maternally derived oocyte proteins which can result in early embryonic developmental arrest^39^. Various types of ubiquitin-mediated modifications are thought to have specific functions. As an ubiquitin ligase, activity of TRIP12 is indispensable for mouse embryogenesis. Gene inactivation of *Trip12* results in embryonic lethality during midgestation^40^. PLAA is required for the Ubiquitin-mediated sorting of membrane proteins from the early to late endosome, targeting them for lysosomal degradation^41^. However, to fully understand its role in oocyte maturation requires further investigation.

The observed differences in gene expression between siRNA injected and noninjected oocytes can be the resultant of three events. Either the presence of dextran that was co-injected to identify correctly injected oocytes changed gene expression; differences could be caused by the physical damage to cell organelles, chromosomes or cell membrane due to the injection procedure; or the interference of the injected siRNA with the genome or mRNA altered gene expression. At this stage it is not possible to exclude one of these events, although it seems highly likely that a cellular response to mechanical injury is associated with changes in gene expression.

We used RNA-seq to identify changes in the transcriptome of single oocytes after non-specific siRNA injection. Our results show that, after microinjection, cattle oocytes are just as capable of maturing to the MII stage during in vitro culture as non-injected oocytes. On the other hand, we detected 43 upregulated and 76 downregulated genes in the injected oocytes, mainly involved in the cytoskeleton, proteasome and ATP synthesis processes. This result demonstrates that there is a ‘background effect’ of microinjecting oocytes and indicates that one should be careful when interpreting data from microinjected oocytes.

## Materials and methods

### Oocyte collection and in vitro maturation

Cattle ovaries were collected from a slaughterhouse in the Netherlands and transported to the laboratory in a polystyrene box at room temperature, within 2 h after slaughter. After washing, the ovaries were maintained in 0.9% NaCl supplemented with 0.1% penicillin/streptomycin (10,000 U/ml; Gibco BRL, Paisley, UK) at 30 °C. Cumulus oocyte complexes (COCs) were aspirated from 2–8 mm follicles^42^. Only oocytes with a multi-layered, compact cumulus complex were selected for microinjection.

### Injection of siRNA into oocytes and in vitro maturation

Microinjection of a commercial, non-specific siRNA: UGGUUUACAUGUCGACUAA (D-001810-01, ON-TARGETplus Non-targeting siRNA #1, Dharmacon, Lafayette, OS, USA) and a mixture of three specific *PIWIL3* siRNAs: GUGACUAGGACAGAAUGGU, GAUACAGUCCAAUCUUACA, GAUGGAAACUCUUUGCUGU (Sigma-Aldrich, Zwijndrecht, the Netherlands) was as described previously^43^. In short, siRNA with a final concentration of 25 μM was mixed with 0.1 mg/ml tetramethylrhodamine (TRITC)labeled 3 kDa dextran (Dextran-TRITC; Molecular Probes, Eugene, OR, USA) in water. For microinjection, 6 denuded GV oocytes were transferred to a 5 μl drop of HEPES buffered M199 + 10% FCS overlaid by mineral oil in a 60 mm dish at 37 °C on an IX71 inverted microscope (Olympus, Leiderdorp, the Netherlands) equipped with a heated stage. 5 μl siRNA mix was loaded into a microinjection needle with a 30° angle and a tip with a 4.3–4.9 μm inner diameter (Eppendorf, Hamburg, Germany). A Femtojet pressure injection system (Eppendorf) was used to inject for 0.2 s at a pressure of 120 hpa. After injection, only oocytes with red fluorescence were selected for culture in 25 µm roscovitine (R7772-1MG, Sigma-Aldrich) in maturation medium without FSH for 16 h in a humidified atmosphere of 5% CO_2_-in-air at 38.5 °C, followed by maturation medium for 22 h (M199 supplemented with 0.02 IU/mL follicle-stimulating hormone [Sioux Biochemical Inc., Sioux Center, IA, USA], 10% FCS, 100 U/ml penicillin and 100 µg/mL streptomycin). Non-injected control oocytes were cultured in the same conditions. After maturation, oocytes with a first polar body were washed in PBS and individually collected into RNase free tubes containing 200 μl RNA lysis buffer and stored at − 80 °C until RNA isolation.

### RNA isolation, reverse transcription and quantitative RT-PCR

Total RNA was extracted using an RNeasy Micro Kit (Qiagen, Valencia, CA, USA) as per manufacturer’s instructions. For cDNA synthesis, 10 μl RNA was mixed with 4 μl 5 × RT buffer (Invitrogen, Breda, The Netherlands), 0.2 μl RNAsin (Promega, Leiden, The Netherlands), 0.75 μl Superscript III reverse transcriptase (Invitrogen), 0.4 μl random primers (Invitrogen), 2 μl DTT (Invitrogen) and 1 μl dNTP (Promega). Reverse transcription took place for 1 h at 55 °C, followed by 5 min at 80 °C before storage at − 20 °C.

The quantitative RT-PCR reactions were performed using a real-time PCR detection system (MyiQ Single-color Real-Time PCR Detection System; Bio-Rad Laboratories, Hercules, CA, USA) with IQ Sybr Green Supermix (Bio-Rad Laboratories). Amplification took place in 40 cycles of 95 °C for 15 s, 30 s at the primer-specific temperature (Supplementary Table 2) and 72 °C for 45 s each. The relative starting quantity was calculated based on the standard curve. Data normalization was performed using *GAPDH* and *SDHA* as reference genes with the same set of samples.

### Single oocyte RNA isolation and Cel-Seq

A total of 24 oocytes (12 non-specific siRNA injected, 12 non-injected, and 12 specific PIWIL3-injected) were collected for total RNA isolation using an RNeasy Micro Kit (Qiagen ) as per the manufacturer’s instructions.

Sequencing library preparation was performed using the CEL-seq2 protocol as described previously^17^ and sequencing was performed on an Illumina NextSeq500 using 75-basepairpaired-end sequencing (Utrecht Sequencing Facility).

Sequencing data were processed as described previously^44^. In brief, after demultiplexing, sequencing reads were mapped to a cDNA library, reads mapping to the same gene with identical UMI were counted as a single read.

### Identification of differentially expressed gene

In order to identify genes with altered expression levels, R package DESeq2 was used. A *P* value was computed based on Poissonian statistics and multiple testing corrected by the Benjamini–Hochberg method^45^. Genes with an adjusted p-value < 0.05 as calculated by DESeq2 were considered to have undergone significant gene enrichment. For identification of up and down-regulated genes in injected oocytes, a Cytoscape App ClueGO was used based on the terms “biological processes”, “cellular components” and “molecular function” in the *Bos taurus* database^46^. Statistical significance was set for a K score of 0.4. The function “GO Term fusion” was selected and GO term restriction levels were set at 1–20, with a minimum of one genes or 4% genes in each GO term。

## Supplementary information

Supplementary file 1.

Supplementary file 2.

## Data Availability

The data discussed in this publication have been deposited in NCBI’s Gene Expression Omnibus^47^ and are accessible though GEO series accession number GSE139867 (https://www.ncbi.nlm.nih.gov/geo/query/acc.cgi?acc=GSE139867).
