# Supplementary material for: Microinjection induces changes in the transcriptome of bovine oocytes
Source: Sci Rep. 2020 Jul 8;10:11211. doi: 10.1038/s41598-020-67603-4 (PMC7343835; doi:10.1038/s41598-020-67603-4)

SUPPLEMENTARY INFORMATION

Microinjection induces changes in the transcriptome of bovine oocytes

Minjie Tan[1], Helena T.A. van Tol[1], Michal Mokry[2], Tom A.E. Stout[3], Bernard A.J. Roelen[3,*]

[1]Department of Population Health Sciences, Faculty of Veterinary Medicine, Utrecht University, Utrecht, the Netherlands.

[2]Epigenomics Facility, University Medical Center Utrecht, Utrecht, the Netherlands

[3]Department of Clinical Sciences, Faculty of Veterinary Medicine, Utrecht University, Utrecht, the Netherlands.

Supplementary Table 1

List of genes differentially expressed between non-injected and scrambled siRNA-injected oocytes, based on an adjusted p value <0.05 with a fold change > 1.2. Presented are the gene expression levels in individual oocytes.

Supplementary Table 2. Primers used for quantitative RT-PCR

**Gene**	**Accession number**	**Sequence (5'-3')**	**Direction**	**Annealing temperature**
PIWIL3	XM_010814123.1	AGAAGGAGCTTCGAGACTGG<br>GATTCTGCTGCAAGGTCAGG	forward<br>reverse	61°C
GAPDH	NM_001034034.2	AGGCCATCACCATCTTCCAG<br>GGCGTGGACAGTGGTCATAA	forward<br>reverse	61°C
SDHA	NM_174178	GCAGAACCTGATGCTTTGTG<br>CGTAGGAGAGCGTGTGCTT	forward<br>reverse	64°C

Supplementary Figure 1. Microinjection of bovine oocytes with *PIWIL3* siRNA mixed with Dextran-TRITC at the germinal vesicle stage and cultured in roscovitine for 16 h before in vitro maturation. A) Brightfield (left) and fluorescence (right) images of matured *PIWIL3* siRNA injected oocytes. Red fluorescence indicates successful injection. Scala bar is 50 µm. B) *PIWIL3* siRNA microinjection does not influence the maturation rate. C) Graph representing the relative *PIWIL3* mRNA expression levels as determined by quantitative RT-PCR in *PIWIL3* siRNA injected oocytes compared with scrambled siRNA injected and non-injected control oocytes. D) Venn diagram showing overlapping and unique transcripts in non-injected, scrambled siRNA injected and *PIWIL3* siRNA injected oocytes.

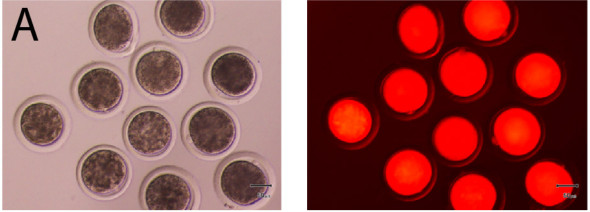

B

	PIWIL3 siRNA	Scrambled siRNA	Non-injected
Oocytes	19	21	24
Matured	16	17	18
Percentage matured	84	81	75

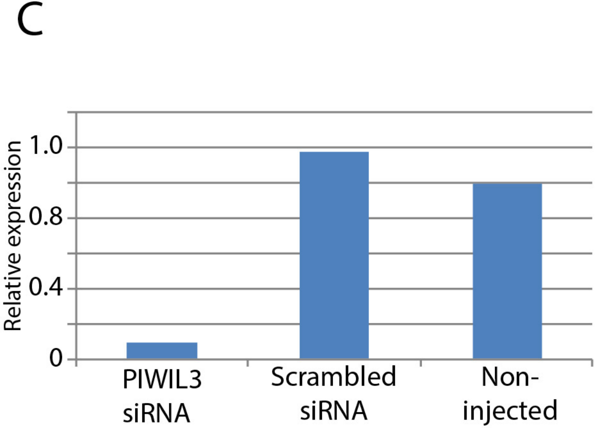

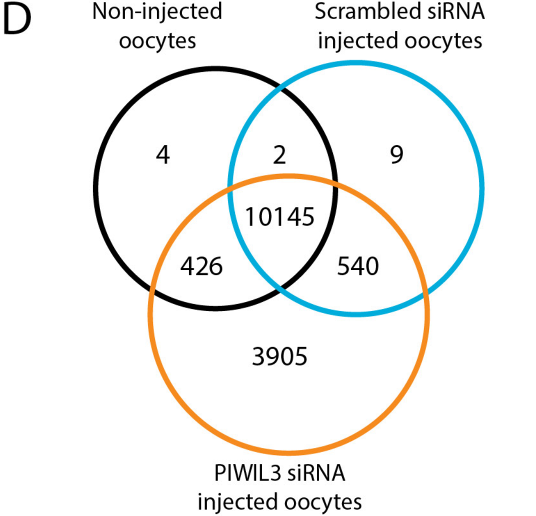

Supplement: Supplementary file 1 [file 41598_2020_67603_MOESM1_ESM.pdf]
